# Higher Fish Intake Is Associated with a Lower Risk of Hip Fractures in Chinese Men and Women: A Matched Case-Control Study

**DOI:** 10.1371/journal.pone.0056849

**Published:** 2013-02-20

**Authors:** Fan Fan, Wen-Qiong Xue, Bao-Hua Wu, Ming-Guang He, Hai-Li Xie, Wei-Fu Ouyang, Su-lan Tu, Yu-Ming Chen

**Affiliations:** 1 Guangdong Provincial Key Laboratory of Food, Nutrition and Health, School of Public Health, Sun Yat-sen University, Guangzhou, People's Republic of China; 2 Guangzhou Orthopaedics Trauma Hospital, Guangzhou, People's Republic of China; 3 Zhongshan Ophthalmic Center, Sun Yat-sen University, Guangzhou, People's Republic of China; 4 Guangdong General Hospital, Guangzhou, Guangdong, People's Republic of China; 5 Orthopaedics Hospital of Baishi District, Jiangmen, Guangdong, People's Republic of China; 6 Sun Yat-sen University Cancer Center, Guangzhou, People's Republic of China; University of Southampton, United Kingdom

## Abstract

**Objectives:**

Fish is rich in nutrients that are favorable to bone health, but limited data are available regarding the relationship between fish intake and hip fractures. Our study examined the association between habitual fish intake and risk of hip fractures.

**Methods:**

A case-control study was performed between June 2009 and June 2012 in Guangdong Province, China. Five hundred and eighty-one hip fracture incident cases, aged 55 to 80 years (mean: 71 years), were enrolled from four hospitals. 1∶1 matched controls by gender and age (±3 years) were also recruited from communities and hospitals. Face-to-face interviews were used to obtain habitual dietary intake and information on various covariates.

**Results:**

Univariate conditional logistic regression analyses showed significantly dose-dependent inverse correlations between the risk of hip fractures and the intake of fresh-water fish, sea fish, mollusca, shellfish, and total fish in all of the subjects (p-trend: <0.001–0.016). After adjusting for covariates, the associations were slightly attenuated but remained significant for all (p-trend: <0.001–0.017) except for fresh-water fish (p = 0.553). The ORs (95%CI) of hip fractures for the highest (vs. lowest) quartile were 0.80 (0.48–1.31) for fresh-water fish, 0.31 (0.18–0.52) for sea fish, 0.55 (0.34–0.88) for mollusca and shellfish, and 0.47 (0.28–0.79) for total fish, respectively. Stratified and interaction analyses showed that the association was more significant in males than in females (*p*-interaction = 0.052).

**Conclusion:**

Higher intake of seafood is independently associated with lower risk of hip fractures in elderly Chinese. Increasing consumption of sea fish may benefit the prevention of hip fractures in this population.

## Introduction

Hip fractures are considered to be the most severe complication of osteoporosis, and have attracted increased attention because of poor prognosis such as leading to chronic pain, increasing the risk of mortality (20–24% patients die in the first year following injury) [1] and disability (40% of survivors lose the ability to walk) [1] and enhancing medical burdens (USD17.14 billion in China in 2011) [2]. The number of incident cases is expected to increase from 1.66 million in 1990 to 4.50–6.26 million per year in 2050 [3]. Given the serious individual and economic consequences of hip fractures, prevention strategies are essential.

Nutritional factors play a key role in the maintenance of optimal bone health [4]. Fish is a major food group worldwide, with a mean intake of 90∼200 g/d (raw weight) consumed in Chinese coastal areas according to the 2002 National Nutritional Survey [5]. Fish is a major source of high-bioavailability protein, n-3 polyunsaturated fatty acids (PUFA), fat-soluble vitamins (such as A and D), and minerals (e.g., calcium, zinc, selenium, and iodine). Many of these nutrients may have beneficial effects on bone health [4]. Some studies have examined the association between fish consumption and bone health, but have generated inconsistent results. Many studies have shown that higher fish or seafood consumption may produce greater bone mineral density (BMD) [6]–[9]. Others have explored the effects of fish consumption on fractures, but the inconsistent results are thought to be the result of various study designs and populations or weak effects [9]–[12]. The majority of these studies, however, were performed in Western populations; thus, limited data have rendered findings on the effects of fish consumption on the risk of bone fractures inconclusive in Asian populations. This study examines the association of habitual intake of total fish and different types of fish with the risk of hip fractures in middle-aged and elderly Chinese in Guangdong Province, a coastal region of China.

## Materials and Methods

### Study population

This 1∶1 matched case-control study was performed between June 2009 and June 2012 in Guangdong Province, China. Eligible cases were required to be hip fracture incident patients within 2 weeks of diagnosis (according to the medical records), confirmed by X-ray image, aged between 55 and 80 years, and living in Guangdong Province for more than 10 years. We attempted to contact all of the eligible patients hospitalized at the following hospitals: Guangzhou Orthopaedics Trauma Hospital, Guangdong General Hospital, First Affiliated Hospital of Sun Yat-sen University, and the Orthopaedics Hospital of Baishi District in Jiangmen city, Guangdong Province. Subjects with the following conditions were excluded: (i) pathological or high-energy fractures (for example, automobile accidents or a fall from above chair height); (ii) substantial changes in dietary habits within the previous 5 years; (iii) chronic disease that might affect dietary habits such as diabetes, stroke, coronary disease, cancer, cognitive disorder, liver cirrhosis, renal failure, thyroid disorder, or chronic diarrhea; (iv) current use of exogenous estrogens exceeding 3 months, corticosteroids, thiazine, or other medications known to affect endocrine balance; and (v) poor vision that might affect routine activities. Each case was matched by one control with the same gender and age (±3 years). Apparently healthy community residents in the same cities and inpatients who had been hospitalized within 1 week with one of the following diseases were accepted as control subjects: influenza, pneumonia, benign ophthalmic or otorhinolaryngologic tumor, and acute surgical disease or cataract in one eye. With the exception of a history of any fracture, the same selection criteria were applied to the control subjects that were applied to the case-patients. Written informed consent was obtained from all participants before the interviews and the ethics committee of the School of Public Health of Sun Yat-sen University approved the study.

### Data collection

Face-to-face interviews were conducted by experienced interviewers with relevant knowledge. We used a structured questionnaire to collect the following information: 1) sociodemographic characteristics (e.g., education, occupation, marital status); 2) lifestyle habits (e.g., smoking, alcohol drinking, tea consumption, physical activity); 3) habitual dietary consumption in the previous 12 months before diagnosis (case-patients) or interview (control subjects); 4) history of chronic diseases and medications; and 5) history of menstruation and child-bearing for female participants. Subjects were also asked whether they had taken calcium or multivitamin supplements daily for at least six consecutive months in the past ten years, and how long they had use the supplements. Each interviewer completed an equal proportion of interviews between case and control groups.

We used a 79-item food-frequency questionnaire (FFQ), validated by a local source population (the correlation coefficient between the FFQ and six 3-day dietary records within 6 months was 0.30 for fish [13]), to assess dietary consumption. The mean intake of food per day, week, month, or year in the year prior to the diagnosis or interview was reported. For seasonal foods, participants were asked to report how many months of the year they consumed each item. Photographs of food portion sizes were provided to help participants estimate the amount of food intake. Fish intake was evaluated by using the following items: (1) freshwater fish (e.g., grass carp, black carp, bullhead, crucian carp, mandarin fish, etc.); (2) sea fish (e.g., pomfret, grouper, golden thread fish, hairtail, etc.); (3) mollusca (e.g., squid, cuttlefish, oyster, scallop); (4) shrimp and crab. Based on the similarities in nutrient composition, we classified seafood into three groups: freshwater fish, sea fish, and mollusca and shellfish. Daily intakes of dietary energy and nutrients were calculated according to the 2002 China Food Composition Table [14].

### Statistical analysis

All data were imputed doubly by two investigators independently using Epidata 3.1. To achieve an approximately normal distribution for the statistical analysis, logarithmic transformation was used in the daily energy intakes, and square root transformation was applied to the other dietary exposures. Dietary intakes were adjusted for daily total energy intake using the residual method. We divided subjects into gender-specific quartiles (Q1–Q4) based on the distribution of dietary intake among the control group, and then applied the gender-specific cutoffs to the cases.

Conditional logistic regressions were conducted, with the bottom quartile group defined as the reference group. Tests were considered statistically significant at p<0.05 (two-tailed), and odds ratios (ORs) with their corresponding 95% confidence intervals (CIs) were yielded in both univariate and multivariate analyses. The ordinal values for the dietary intake categories were included as continuous variables in linear trend tests. Demographic characteristics and other potential osteoporosis risk factors were evaluated by paired *t*-tests (continuous variables) or paired *χ^2^* tests (categorical variables), which, with p≤0.05, would be induced into the multivariate models as confounders by the forward stepwise method. The intakes of other food groups (cereals, soybeans, fresh vegetables, fruits, dairy products, nuts, and livestock and poultry meat) and total energy were also included in the multivariate adjustments.

Stratified analyses were conducted according to gender and the sources of control. Women's menstrual histories (years since menopause and oral contraceptive and estrogen use) were adjusted for in the multivariate analysis. Multiplicative interactions were estimated by the likelihood ratio test.

## Results

Table 1 shows the process of participant selection. 581 cases with 581 matched controls were analyzed. Of the 581 cases, 396 had femoral neck fractures while the other 185 had intertrochanteric fractures. Of the 581 controls, 398 were recruited from communities and 183 were recruited from hospitals.

**Table 1:** Process of participant selection.

	Cases	Matched controls	
		Community	Hospital
Screened	1137	708	307
Excluded (in total)	556	310	124
Diseases affecting dietary habits	246	154	52
Diseases affecting routine activities	133	96	22
Pathological or high-energy fractures.	44	0	0
Unable to communicate.	23	8	10
Refused to participate	94	0	20
Unreasonable energy intakes*	16	5	7
History of any fracture	0	47	13
Included in the analyses	581	398	183

* Reasonable range: 800–4000 kcal/d for males and 500–3500 kcal/d for females.

The cases and controls had similar gender distributions due to the paired design. The controls were more likely than the case patients to have a higher BMI, education level, and household income in addition to the following: no family history of fracture, engaging in mental work, drinking tea, being married, increased physical activity, and the consumption of multivitamin and calcium supplements. Female case-patients tended to exhibit less physical activity, more years since menopause, and less use of oral contraceptives or estrogen than their matched controls (Table 2).

**Table 2:** Demographics, lifestyle characteristics, and select hip fracture risk factors of study population in Guangzhou, China.

	Men (148 pairs)			Women (433 pairs)		
	Cases	Controls	p	Cases	Controls	p
Age, *y*	70.03±6.96	69.49±6.99	***0.016***	71.37±6.62	71.39±6.48	0.898
Body mass index, *kg/m^2^*	20.93±2.11	23.21±2.36	***<0.001***	21.40±3.90	22.93±3.07	***<0.001***
Marital status, *N(%)*			***0.006***			***<0.001***
Married	116(78.4)	130(88.4)		230(53.6)	296(69.0)	
Unmarried/Divorced/Widowed	32(21.6)	17(11.6)		199(46.4)	133(31.0)	
Education level, *N(%)*			***0.001***			***<0.001***
Primary school or below	80(54.1)	48(32.7)		277(64.9)	209(48.9)	
Secondary school	23(29.6)	36(29.4)		42(9.8)	81(19)	
High school or above	45(30.4)	63(42.9)		108(25.3)	137(32.1)	
Occupation^a^, *N(%)*			***0.022***			***0.002***
Full mental work	31(20.9)	36(24.3)		64(14.8)	80(186)	
Main mental work	28(18.9)	48(32.4)		64(14.8)	99(23.0)	
Main physical labor	33(22.3)	25(16.9)		72(16.7)	60(13.9)	
Full physical labor	49(33.1)	36(24.3)		208(48.3)	153(35.5)	
Other	7(4.7)	3(2.0)		23(5.3)	39(9.0)	
Household income, *Yuan/month/person*, *N(%)*			***0.004***			***<0.001***
≤500	7(4.8)	2(1.4)		35(8.1)	10(2.3)	
501∼2000	65(44.2)	40(27.0)		197(45.8)	147(34.2)	
2000∼3000	54(36.7)	79(53.4)		147(34.2)	195(45.3)	
>3000	21(14.3)	27(18.2)		51(11.9)	78(18.1)	
Social status, *N(%)*			***0.008***			***0.001***
Bad	44(29.7)	23(15.5)		103(23.9)	61(14.1)	
General	54(36.5)	67(45.3)		206(47.8)	234(54.0)	
Good	50(33.8)	58(39.2)		122(28.3)	138(31.9)	
Family history of fractures, *N(%)*			0.087			0.137
Father	9(6.1)	5(3.4)		17(3.9)	12(2.8)	
Mother	13(8.8)	5(3.4)		53(12.3)	36(8.3)	
Orientation of house^b^, *N(%)*			***0.043***			0.211
Exposure to the sun	122(82.4)	129(90.2)		337(78.9)	321(75.2)	
Smoking status^c^, *N(%)*	69(46.6)	54(36.5)	0.077	17(3.9)	7(1.6)	0.064
Passive smoking^d^, *N(%)*	45(30.4)	20(13.5)	***<0.001***	95(22.0)	76(17.6)	0.081
Alcohol drinker^e^, *N(%)*	28(18.9)	18(12.2)	0.143	10(2.3)	16(3.7)	0.327
Tea drinker^f^, *N(%)*	60(40.5)	86(58.1)	***0.003***	139(32.2)	169(39.1)	***0.042***
Calcium supplement user, *N(%)*	18(12.2)	39(26.4)	***0.001***	134(30.9)	171(39.5)	***0.006***
Multivitamin user, *N(%)*	11(7.4)	39(26.4)	***<0.001***	36(8.3)	103(23.8)	***<0.001***
Physical activity^g^, *MET• h/d*	69.01±47.91	71.28±43.59	0.624	76.35±45.30	88.36±64.37	***0.001***
Years since menopause, *y*				22.48±7.56	21.19±8.96	***0.001***
Oral contraceptive user, *N(%)*				25(6.0)	76(18.3)	***<0.001***
Estrogen user, *N(%)*				7(1.7)	42(10.1)	***<0.001***

Continuous variables were described by means ±standard deviation.

a Occupation: “mental work” refers to those works which need less physical activity, such as administrators, managers, clerks, professionals or other white collars.

b House orientations: ‘head’ referred to the orientation of the living room. Housing with east, south, southeast, southwest, northeast, and northwest orientations designated a head in the sun and other orientations designated a head in the shade.

c Smoking was defined as having smoked ≥1 cigarette daily for at least six consecutive months.

d Passive smoking was defined as being exposed to other's tobacco smoking for ≥5 minutes daily in the previous five years.

e Alcohol drinkers were defined as having had wine ≥1 time(s) daily for at least six consecutive months.

f Tea drinkers were defined as drinking at least one cup of tea per week in the previous six months.

g Physical activities included daily occupational, leisure-time, and household-chores, evaluated by metabolic equivalent (MET) hours per day.

The mean intakes of main food groups are shown in Table 3. In the control group, the mean values for the energy-adjusted consumption of freshwater fish from quartile 1 to quartile 4 were 2.69, 10.90, 17.86, and 39.10 (g/d) in males and 3.00, 10.49, 20.76, and 55.81 (g/d) in females, while the mean values for sea fish in the same quartiles were 0.54, 3.05, 6.47, and 35.07 (g/d) in males and 0.12, 1.40, 6.10, and 26.93 (g/d) in females.

**Table 3:** Intake of main food groups for study population in Guangzhou, China^a^.

	Men (148 pairs)		*p*	Women (433 pairs)		*p*
	Cases	Controls		Cases	Controls	
Dietary energy, *kcal/d*	1388±345	1402±385	0.668	1274±381	1323±360	**0.044**
Cereals	699±121	689±148	0.513	656±134	630±136	***0.001***
Soy protein	3.11±3.99	4.67±4.71	***0.001***	2.36±3.06	3.44±3.73	***<0.001***
Vegetables	232±106	289±130	***<0.001***	241±120	288±130	***<0.001***
Fruits	39.8±30.6	66.6±56.8	***<0.001***	52.7±42.9	67.3±57.7	***<0.001***
Milk and dairy products	46.7±71.6	77.1±101.9	***0.002***	71.6±113.4	95.4±94.7	***<0.001***
Nuts	9.28±10.52	12.42±13.71	***0.023***	6.75±11.03	11.66±16.65	***<0.001***
Meat	87.7±51.9	63.2±31.5	***<0.001***	80.2±52.7	64.0±39.6	***<0.001***
Poultry	22.6±16.5	17.8±12.3	***0.004***	19.0±15.0	19.6±16.8	0.629
Total fish	26.2±22.9	34.5±26.5	***0.002***	26.4±22.3	34.6±33.3	***<0.001***
Freshwater fish	16.4±15.3	17.6±17.2	0.490	17.0±16.1	22.5±32.0	***0.001***
Sea fish	4.94±11.64	11.28±17.92	***<0.001***	5.72±12.00	8.62±13.75	***0.001***
Mollusca and shellfish	4.57±7.97	5.57±8.93	0.200	3.39±6.98	3.71±5.94	0.463

Continuous variables were described by means ±SD and evaluated by t-test.

a Mean intakes of food groups were adjusted for daily energy intake using the residual method. The mean of daily energy intake was 1355 kcal (male) or 1278 kcal (female).

Univariate conditional logistic regression analyses showed significantly dose-dependent inverse associations between the intake of total fish and all fish subtypes and the risk of hip fracture in all of the subjects (p-trend: <0.001–0.016). After adjusting for age, BMI, socioeconomic factors, physical activity, passive smoking, tea drinking, and dietary factors, significant associations remained for total fish and other fish subtypes, with the exception of freshwater fish. Compared to the lowest quartile, the ORs (95%CI) of hip fractures for the highest quartile were 0.80 (0.48–1.31) for fresh-water fish, 0.31 (0.18–0.52) for sea fish, 0.55 (0.34–0.88) for mollusca and shellfish, and 0.47 (0.28–0.79) for total fish in the multivariate model, respectively (Table 4).

**Table 4:** Odds ratio (95% CIs) of hip fractures for quartiles of seafood intake in Guangzhou, China.

	Quartiles of dietary energy-adjusted intake				P for trend
	1 (referent)	2	3	4 (highest)	
Freshwater fish					
Intake (M/F), *g/d*	2.69/3.00	10.90/10.49	17.86/20.76	39.10/55.81	
N (cases/control)	180/145	130/146	155/145	116/145	
OR I (95%CI)	1.00	0.70(0.51–0.98)	0.82(0.59–1.15)	0.60(0.42–0.86)	***0.016***
OR II (95%CI)	1.00	0.98(0.63–1.54)	1.19(0.74–1.90)	0.80(0.48–1.31)	0.553
Sea fish					
Intake (M/F), *g/d*	0.54/0.12	3.05/1.40	6.47/6.10	35.07/26.93	
N (cases/control)	224/145	159/146	119/145	79/145	
OR I (95%CI)	1.00	0.69(0.51–0.95)	0.49(0.35–0.69)	0.31(0.22–0.46)	***<0.001***
OR II (95%CI)	1.00	0.64(0.41–1.00)	0.46(0.29–0.72)	0.31(0.18–0.52)	***<0.001***
Mollusca and shellfish					
Intake (M/F), *g/d*	0.27/0.08	1.83/0.73	4.15/2.88	16.04/11.15	
N (cases/control)	211/145	145/145	112/146	113/145	
OR I (95%CI)	1.00	0.70(0.51–0.95)	0.49(0.35–0.70)	0.50(0.35–0.71)	***<0.001***
OR II (95%CI)	1.00	0.62(0.41–0.96)	0.49(0.30–0.78)	0.55(0.34–0.88)	***0.004***
Total fish					
Intake (M/F), *g/d*	9.75/7.88	22.85/20.95	35.25/36.33	70.15/73.42	
N (cases/control)	196/145	152/145	145/146	88/145	
OR I (95%CI)	1.00	0.75(0.54–1.03)	0.66(0.47–0.93)	0.40(0.28–0.85)	***<0.001***
OR II (95%CI)	1.00	0.89(0.57–1.38)	1.04(0.64–1.67)	0.47(0.28–0.79)	***0.017***

Intake (M/F): Mean intake of seafood in male/female controls.

OR I, OR II: from conditional logistic model. OR I: without further adjustment; OR II: adjusted for BMI, education, marital status, occupation, household income, social status, house orientation, family history of fractures, passive smoking, tea drinking, calcium supplement user, multivitamin user, physical activity, daily energy intake, and energy-adjusted intakes of other food groups (including cereals, soybeans, vegetables, fruits, fresh meats, fresh poultry, nuts, milk and dairy products) by stepwise forward method. 95%CI: 95% confidence interval.

Stratified and interaction analyses demonstrated that the effect of sea fish intake was much more significant in males than in females (OR for Q4 vs. Q1: 0.10 vs. 0.36, *p* interaction: 0.052). The associations of total fish and its subtypes with hip fracture did not differ in subgroups stratified by control sources (Table 5).

**Table 5:** Odds ratio (95% CIs) of hip fractures for quartiles of seafood stratified by gender, source of controls, and fracture sites in Guangzhou, China.

		Quartiles of dietary energy-adjusted intake				*p*-trend	*p*-intera -ction
Variable	Pair N	1 (referent)	2	3	4 (highest)		
Freshwater fish							
Gender							0.189
Male	148	1.00	1.09(0.19–6.38)	1.82(0.29–11.38)	2.30(0.41–12.82)	0.270	
Female	433	1.00	0.82(0.47–1.43)	1.08(0.60–1.95)	0.52(0.28–0.97)	0.212	
Source of controls							0.143
Community	398	1.00	1.07(0.56–2.03)	1.76(0.90–3.45)	1.21(0.60–2.46)	0.510	
Hospital	183	1.00	1.35(0.64–2.87)	1.01(0.47–2.19)	0.60(0.26–1.38)	0.215	
Sea fish							
Gender							0.052
Male	148	1.00	0.24(0.06–1.02)	0.03(0.01–0.19)	0.10(0.02–0.47)	*0.015*	
Female	433	1.00	0.86(0.51–1.48)	0.72(0.40–1.29)	0.36(0.18–0.69)	*0.003*	
Source of controls							0.768
Community	398	1.00	0.47(0.25–0.90)	0.34(0.17–0.68)	0.29(0.14–0.58)	*<0.001*	
Hospital	183	1.00	0.75(0.35–1.60)	0.30(0.13–0.68)	0.18(0.06–0.54)	*<0.001*	
Mollusca and shellfish							
Gender							0.540
Male	148	1.00	0.51(0.09–2.74)	0.89(0.17–4.61)	0.08(0.01–1.37)	*0.252*	
Female	433	1.00	1.00(0.59–1.69)	0.47(0.26–0.87)	0.59(0.32–1.06)	*0.016*	
Source of controls							0.403
Community	398	1.00	0.37(0.20–0.69)	0.39(0.20–0.77)	0.59(0.31–1.12)	*0.039*	
Hospital	183	1.00	0.54(0.25–1.17)	0.34(0.15–0.79)	0.25(0.09–0.68)	*0.002*	
Total fish							
Gender							0.864
Male	148	1.00	1.24(0.29–5.41)	2.10(0.41–10.73)	0.41(0.07–2.43)	0.665	
Female	433	1.00	1.06(0.61–1.86)	1.18(0.65–2.12)	0.42(0.22–0.78)	*0.037*	
Source of controls							0.389
Community	398	1.00	0.86(0.50–1.66)	1.19(0.58–2.44)	0.57(0.28–1.15)	0.200	
Hospital	183	1.00	0.95(0.47–1.91)	1.36(0.63–2.91)	0.25(0.09–0.70)	0.075	

Odds ratios (95% CI): from multivariate conditional logistic regression models. Covariates adjusted for: see ORII in Table 3. For women, years since menopause, oral contraceptive user, estrogen user were further adjusted for by the stepwise forward method.

## Discussion

This matched case-control study, with 581 cases and 581 controls, revealed that the intake of fish and shellfish had a statistically significant protective effect on the risk of hip fracture. The favorable effect of sea fish was much stronger than that of freshwater fish. Fish are a major source of animal food in the traditional diet among the coastal regions of mainland China. Our finding suggests that an increase in fish consumption, especially sea fish, may benefit the prevention of hip fractures in this population.

Most but not all previous studies have supported the hypothesis that a greater intake of fish is favorable for the prevention of osteoporosis. A large population-based study in China by Zalloua et al. [6] found that increasing seafood consumption (>250 vs. <250 g/week) was significantly associated with greater BMD at the total body and hip in women (p<0.01). Farina et al. [7] found that high intakes (≥3 vs. <3, servings/wk) of fish were associated with the maintenance of femur neck BMD in both men and women in their 4-year Framingham Osteoporosis Study. Similar results were observed in cross-sectional studies in Hong Kong postmenopausal women [8] and in 1,305 participants aged ≥65 years from a U.S. cardiovascular health study [9]. However, fracture studies have produced inconsistent results. In the Nurses' Health Study involving 72,337 post-menopausal women, a dose-response relationship was noted between a higher intake of dark fish (sea fish) and lower risk of hip fracture (*p*-trend = 0.03) [10]. A similar result was observed in a case-control study of hip fractures (294 cases and 498 controls) in Japan [11], but not in a cohort study with a 12-year follow up in 4,573 people in Japan [12] or in the Cardiovascular Health Study with 5,045 participants and an 11-year follow up [9] possibly due to limited study size. Generally consistent with the majority of previous studies, our findings showed that greater intakes of sea fish and shellfish had a protective effect against the risk of hip fracture.

The positive effects of fish can be partially attributed to rich nutrients that support bone health. Fish and shellfish are good sources of high-bioavailability protein, which many studies have found may positively affect bone health by improving calcium absorption, increasing insulin-like growth factor I, and improving lean body mass [15]. Other researchers have proposed that excess protein may have an adverse effect on bone by increasing acid load [16], [17]. The potential renal acid load (PRAL) values (mEq/100 g) in sea fish (8.29) and freshwater fish (8.25) were slightly lower than those in pork (9.9) and beef (10.0) [14], [18]. The beneficial effects of seafood may be due to the high-bioavailability protein it contains and a relatively lower acid-load generated by the displacement of meat protein. Another important nutrient that may play a role in this association is calcium. Calcium has been considered the key nutrient for bone health. A meta-analysis of eighteen studies reported a positive association between dietary calcium and risk of hip fracture (OR = 0.96, 95% CI: 0.93–0.99) [19]. The level of calcium in fish was much higher than in other meat [14]. Thus, the positive effect of seafood might be partially explained by its high calcium content.

We found that the favorable effect of sea fish on hip fracture was more substantial than that of freshwater fish, as was observed in a population-based cross-sectional study on fish and BMD in Hong Kong [8]. The beneficial effect was reported in sea fish but not in fresh-water fish on BMD in Chinese women in the cross-sectional study [6] and on the incidence of hip fracture in the Nurses Health Study cohort [10]. Why sea fish have a more favorable effect on bone health than freshwater fish remains uncertain. The differential effect between sea fish and freshwater fish is unlikely to be explained by high-quality protein and calcium, because their presence in the two types of fish was similar. Vitamin D and n-3 polyunsaturated fatty acid might partially explain the difference between sea fish and freshwater fish. Sea fish are richer in vitamin D than freshwater fish and vitamin D has long been thought to enhance the effects of calcium on bone health [20]. The human body most commonly obtains vitamin D from the diet or skin exposure to sunlight. However, the endogenous production of vitamin D tends to decrease with age and elderly people become more dependent on dietary sources to maintain adequate vitamin D status [21]. Second, sea fish are richer sources than freshwater fish of the long-chain n-3 fatty acids eicosapentaenoic acid and docosahexaenoic. Many studies have shown the beneficial effects of n-3 fatty acids on bone health in both humans and animals [22]–[24]. The possible mechanisms for the beneficial effects of dietary n-3 fatty acids on bone are extensive and include increasing calcium absorption [25], [26], increasing the synthesis of bone collagen [27], inhibiting bone resorption [25], decreasing urinary calcium excretion [27], and modulating the action of inflammatory cytokines, e.g., interleukin-1, interleukin-6, and tumor necrosis factors [28]–[30]. Thus, the varied effect of both types of fish on bone health might be partially explained by the different content of vitamin D and n-3 fatty acids in the population.

We explored whether the beneficial effects of sea fish varied with gender, control sources, or fracture sites. The association between the intake of sea fish and the risk of hip fracture was more substantial in males than in females (OR, comparing extreme quartiles: 0.10 vs. 0.36, *p*-interaction: 0.052). This difference might stem from a large between-individual variation in the sea fish intakes of males and females (mean difference of intake between Q4 and Q1 in males vs. females, g/d: 34.53 vs. 26.81).

Our study has the following limitations. First, the time sequence between the exposure and outcome may be uncertain in the case-control study. However, it is unlikely to have an inverse time sequence because (i) only new cases were selected, (ii) we excluded both cases and controls with chronic diseases that might change dietary habits or nutritional factors, and (iii) adults generally maintain a long-term stable dietary habit [31], [32]. Second, hospital-based case-control studies are prone to selection biases. We controlled the selection bias by using multiple cases sourced from four different hospitals and controls from various hospitals and the community. The stratified analysis showed similar associations between hospital- and community-based controls. Third, while it is difficult for elderly participants to accurately recall habitual dietary intake, a differentially biased report of the fish intake between cases and controls is unlikely because the beneficial effects of fish consumption on bone health is not publically confirmed or well-known. We also controlled the interviewer bias through standardized training and by having the interviewers conduct an equal proportion of case and control interviews. Finally, apart from age and gender, other socioeconomic factors and food intakes were not balanced between the cases and controls. Thus, we used a multivariate model to control the possible confounders.

### Conclusions

Our findings show that a higher intake of seafood is significantly and independently associated with a lower risk of hip fracture in the elderly. Therefore, increasing sea fish consumption may benefit the prevention of hip fracture in the elderly.
